# Evaluation of BASE eConsult Manitoba: patient perspectives on the use of electronic consultation to improve access to specialty advice in Manitoba

**DOI:** 10.1186/s12913-022-08913-3

**Published:** 2023-02-09

**Authors:** Alexander Singer, Laurie Ireland, Zahra Sepehri, Kelly Brown, Kevin Turner, Clare Liddy, Erin Keely, Luis Oppenheimer

**Affiliations:** 1 Department of Family Medicine, Max Rady College of Medicine, Rady Faculty of Health Sciences, University of Manitoba, D009-780 Bannatyne Ave., MB Winnipeg, R3E 0W2 Canada; 2 Department of Family Medicine, University of Manitoba, D009-780 Bannatyne Ave., MB Winnipeg, R3E 0W2 Canada; 3 Nine Circles Community Health Centre, 705 Broadway Ave. , Winnipeg, MB R3G 0X2 Canada; 4 Department of Internal Medicine, Zabol University of Medical Sciences, Zabol, Iran; 5 Department of Family Medicine, Max Rady College of Medicine, University of Manitoba, P219 – 780 Bannatyne Ave, MB Winnipeg, R3E 0W2 Canada; 6 CancerCare, Winnipeg, MB Canada; 7 eConsult Centre of Excellence, University of Manitoba Alumni, Winnipeg, MB R33 0W2 Canada; 8 7 C.T. Lamont Primary Health Care Research Centre, Bruyere Research Institute, Ottawa, ON Canada; 9 Department of Family Medicine, University of Ottawa, Ottawa, ON Canada; 10 eConsult Centre of Excellence, The Ottawa Hospital, Ottawa, ON Canada; 11 Department of Medicine, University of Ottawa, Ottawa, ON Canada; 12 Division of Endocrinology and Metabolism, The Ottawa Hospital, Ottawa, ON Canada; 13 Department of Surgery, University of Manitoba and Winnipeg Regional Health Authority, Winnipeg, MB Canada

**Keywords:** Access to care, Primary care, Qualitative research, Quality of care, eConsult

## Abstract

**Background:**

The burden of waiting to access specialist expertise may contribute to poorer health outcomes and causes distress for patients and providers. One solution to improve access to specialist care is to use innovative tools such as remote asynchronous electronic consultation (eConsult). Modeled after the Champlain BASE™ (Building Access to Specialist Advice) eConsult service, BASE™ eConsult Manitoba was launched in 2017 to help address long waits for patients to access specialist advice.

**Objective:**

We aimed to evaluate patients’ experiences after obtaining a BASE™ eConsult Manitoba service in their primary care setting.

**Methods:**

Patients whose Primary Care Providers (PCPs) used BASE™ eConsult as part of their care were asked to participate and complete a telephone-based or online 29-question survey between January 2021 and October 2021. The survey questions were created in consultation with patient partners and based on questions asked in studies done in other jurisdictions.

**Results:**

Of the 36 patients who chose to participate, 29 completed the entire survey (80%). Two-thirds (*n* = 22) agreed that eConsult has been helpful in their situation, and over 80% (*n* = 24) of participants agreed that eConsult was an acceptable way to access specialist care. During the visit when their PCP sent the eConsult, 7 patients were expecting to be referred to a specialist for a face-to-face consultation. Over half of all respondents (*n* = 15) reported that before the eConsult occurred, their PCP asked them what questions they wanted to be answered by the specialist. Almost all of these respondents’ questions were fully answered by the eConsult. All of the respondents were satisfied with the experience of receiving an eConsult.

**Conclusion:**

Using eConsult is an acceptable way to improve access to specialist advice from patients’ perspectives. Consideration should be given to expanding the use of eConsult services to improve access to specialist expertise for PCPs and their patients.

**Supplementary Information:**

The online version contains supplementary material available at 10.1186/s12913-022-08913-3.

## Introduction

Long wait times for patients to access care is a major problem in many healthcare systems. The burden of waiting is significant for patients and may contribute to poorer health outcomes [1–3]. In a survey by the Commonwealth Fund, Canada was shown to have the second longest wait times for seeing a specialist physician compared to 11 other similar health systems [4]. Also, in 2021, the longest-ever waiting time was recorded in Canada [5]. In Manitoba, with a population density of 2 persons per square kilometer compared with the national density of 4.23 persons per square kilometer, the median wait time from referral by a primary care provider to treatment has increased from 10.5 weeks in 1993 to 31.5 weeks in 2021 [3, 6]. Long wait times contribute to patient anxiety and stress, increased pain and suffering, delays in diagnosis, duplication of testing, and potential further deterioration of their conditions, leading to higher costs and reduced satisfaction for patients and providers [2, 3].

One solution to improve access to specialist expertise is using innovative services such as electronic consultation (eConsult). eConsult connects primary care providers (PCPs) with questions to advice from specialists via asynchronous electronic means. In Canada, the Building Access to Specialist Advice (BASE) eConsult model was first adopted in the Champlain district surrounding Ottawa in 2009. The Champlain BASE™ eConsult service is an asynchronous communication platform supporting direct communication between PCPs and specialists. It uses a secure web-based portal where PCPs use a four-field template, including a box for a question regarding patient care directed to a specific specialty service. Usually, these questions address the issues that would have required an in-person referral. The template requires minimal demographic information (i.e. date of birth, and gender). PCPs can attach additional information (such as test results, images). The BASE eConsult model has been spread and scaled up in several other Canadian jurisdictions [7] and has demonstrated effectiveness in reducing wait times, achieving high levels of patient and provider satisfaction, and lowering costs of care [8–11]. A review of similar international eConsultation models has shown similar benefits [12–14].

The BASE eConsult model was launched in Manitoba, Canada, in 2017. The service has grown substantially since its inception and now includes 59 unique specialty services that provide advice to over 366 PCPs. As of October 2022, over 6833 eConsults have been provided in Manitoba, with an average response time of fewer than 5.3 days. At the end of every eConsult, PCPs must complete a mandatory survey that provides the research team with the evaluative data. Survey responses from users indicate that 49% of eConsults in Manitoba avoided the need for an in-person consultation when a referral was initially intended [7].

Prior to this study, patient feedback and perspectives on using the BASE eConsult Manitoba has not been measured. Other jurisdictions have evaluated patient feedback, including a qualitative study by Joschko et al. that explored patients’ perspectives on receiving care through BASE eConsult in Ontario, Canada [15]. The Ontario study reported that patients found BASE eConsult useful in their case, that it was an acceptable way to access specialist care and that they would ask their PCP to use eConsult on their behalf in the future [15].

Another study by Keely et al. explored patients’ perspectives on wait times and using eConsult as an alternative to a face-to-face visit. Most patients thought wait times exceeded what they thought was ideal. About 46% of these patients thought an eConsult would be an appropriate alternative to an in-person specialist visit [16].

In this study, we sought to evaluate patients’ experiences with the use of an asynchronous electronic consultation service (BASE eConsult Manitoba) in selected primary care settings in Manitoba.

## Methodology

This study utilized survey data to gather limited demographic information and patient feedback on their experience of having received an electronic consultation (Additional file 1: Appendix A. Patient Survey questions). The patient survey questions were developed by our research team in collaboration with our patient advisors and based on a similar patient survey that was co-developed by patient advisors from across Canada as part of the Connected Medicine workshop organized by the Canadian Foundation for Healthcare Improvement (CFHI) in 2017 [17]. Our patient advisors were included in the initial discussions regarding the design and implementation of the study. Our patient advisors also attended the monthly study meetings which included discussion of this and other BASE eConsult Manitoba activities. Their feedback led to several changes and improvements in the survey questions and supported our interpretation and analysis. Four of our survey questions are identical to those used in the Ontario patient perspective survey on the BASE eConsult service conducted by Joschko et al. to allow for direct comparison [15].

### Participant recruitment

Clinics using the BASE eConsult Manitoba Service in 2020 were invited to participate in recruitment. Clinics that participated in the patient survey distribution were located in an urban setting (Winnipeg). Our original protocol had sought to include a clinic in Northern Manitoba, but there were administrative challenges and they were not able to participate in distributing surveys. Clinic leads received an email invitation to participate in recruiting patients (details explained in Additional file 2: Appendix B. Clinic Invitation to participate). Targeted clinic recruitment also took place by sending follow-up emails to invite clinics with the highest volume of BASE eConsult Manitoba service. Where required, regional or organizational approvals were obtained for clinics to participate.

Patient recruitment was initiated by generating a query in the participating clinic’s Electronic Medical Record to identify patients who had received a BASE eConsult related to their care. The inclusion criteria were any patient who had received an eConsult since the inception of the service. Clinic administrative staff or providers were supported by the research team to develop the query to identify potential participants if required.

Potential participants were mailed a paper letter (Additional file 3: Appendix C. Patient invitation to participate) or received an email message directly from their clinic inviting them to participate in the Patient Survey. The request to complete the survey was sent to the patients only once. Patients were invited to call the research coordinator to learn more or to participate in the study’s survey by phone. Alternatively, a link was provided in the paper letter or email for patients to participate in the survey online through SurveyMonkey. A consent disclosure statement preceded the survey either over the phone or on the SurveyMonkey site based on the patient’s choice of how to participate. We excluded any patients who did not consent to complete the survey. Patients were instructed in the invitation letter that they had four months to respond. Patient participants who responded to the link or via phone were offered a small honorarium of $10.00 cash or gift card.

### Data analysis

The age and clinic location of respondents were collected. Patient satisfaction with the BASE eConsult Manitoba service was analyzed using a Likert scale. Yes/No survey responses were analyzed to tabulate the percentage of responses. For qualitative responses, participants’ perceptions of the eConsult service were gathered and coded manually using inductive coding. A member of the research team (research coordinator) read a random sample of data. Codes were created to cover the sample. The sample was reread, and codes were applied. Notes were made where codes did not match or additional codes were needed. New codes were created based on the rest of the responses. All the responses were recoded again. This process was repeated until all of the data had been coded. The same process was done by a research assistant. Then they exchanged the raw data to code them independently to ensure accuracy. They compared their results and met regularly to reach a consensus. A flat coding frame was used to assign the same level of specificity and importance to each code.

Analysis was conducted using Microsoft Excel.

## Results

Thirty-six patients chose to participate in the study. Four participants completed the survey by phone, and 32 chose to do the online survey through SurveyMonkey. The research coordinator entered the answers of the four participants in SurveyMonkey and added a note indicating the data entered by her. Overall, Seven participants just answered the first question asking if they were interested in participating in the study. They agreed to participate; but did not answer any other questions. 29 completed questions in the survey (83%). The demographic characteristics of the patients are shown in Table 1. The minimum and maximum of the age of the participants were 15 and 97 years old, respectively.

**Table 1:** Demographic characteristics of the patients who participated in the SurveyMonkey in Manitoba (*n* = 29)

**Characteristics**	**N**	**%**
Age		
0–16	1	3.44
17–29	2	6.89
30–49	10	34.48
50–65	11	37.39
+65	5	17.26

Of the 29 patients who completed the survey, 76% agreed that eConsult has been useful in their situation, 83% agreed that eConsult was an acceptable way to access specialist care, and 55% thought that the eConsult service is an acceptable alternative to face-to-face specialist consultations. 66% of respondents responded that they would ask their PCP to utilize the eConsult service on their behalf again in the future. Table 2 shows the participants’ perspective on eConsult services in Manitoba.

**Table 2:** Participants’ perspective on eConsult services in Manitoba between January 2021 and October 2021 (*n* = 29)

Characteristic	Survey Responses	
	N	%
Do you think that the eConsult service was useful in your situation?		
Yes	22	76
No	2	7
Unsure	5	17
Do you think that the eConsult service is an acceptable way to access specialist advice?		
Yes	24	83
No	1	3
Unsure	4	14
Do you think that the eConsult service is an acceptable alternative to face-to-face specialist consultations?		
Yes	16	55
No	4	14
Unsure	8	28
Unanswered	1	3
Would you ask your Primary Care Provider to use the eConsult service on your behalf in the future?		
Yes	19	66
No	2	7
Unsure	5	17
Unanswered	3	10

During the visit when their PCP sent the eConsult, 24% (*n* = 7) were expecting to be referred to a specialist for a face-to-face consultation and 22 respondents did not expect to be referred. Over half of (51%) all respondents (*n* = 15) reported that before the eConsult occurred, their PCP asked them what questions they wanted answered by the specialist. 14 out of 15 of the respondents said the questions they had, were answered fully by the eConsult. One patient (1 out of 15) had not received the eConsult results when they participated in the survey.

Six patients (21%) answered “No” to the question “Before the eConsult occurred, did your Primary Care Provider ask you what questions you wanted answered by the specialist?” and 8 respondents said that they were unsure.

At the time of the study, about half (*n* = 14) of the respondents (*n* = 26) had received the results from their eConsult (54%). Of these 14 respondents, half of them received their results in less than one week, and two of them received the results in 7 days. Two of them received the results in 14 days, and two patients received it in 30 days. One of the respondents was not sure about the time. These patients unanimously agreed that this wait time was acceptable to them.

Of those who did not receive their results (*n* = 6), almost all of them (23%) stated that a wait time less than one week would be more acceptable.

**Fig. 1:**
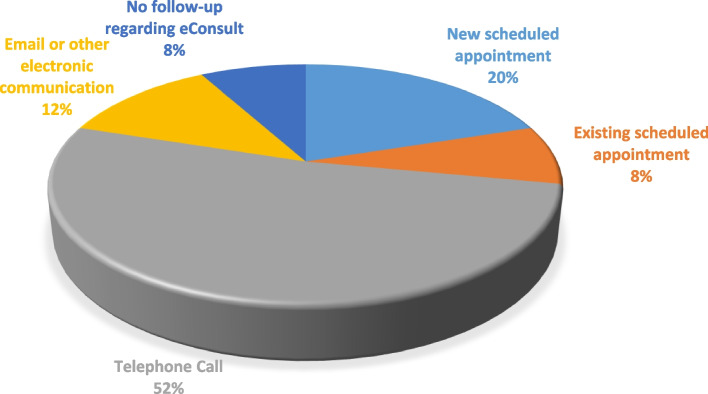
Different ways the eConsult advice was provided to the patients in Manitoba

Figure 1 shows how the eConsult advice was provided to the patients. Overall 25 patients answered to this question. Telephone calls was the most common way (13 of 25).

Furthermore, we aimed to describe other benefits or harms perceived by patients with receiving specialty advice through electronic consultation. After receiving an eConsult, 65% (*n* = 19) of participants avoided an in-person visit to a specialist. Those who avoided a specialist in-person visit, mentioned different advantages such as avoided costs (travel, parking, child or eldercare) for 69% (*n* = 13), avoided time off work for 63% (*n* = 12), reduced stress level or anxiety for 47% (*n* = 9), avoided potential exposure to COVID-19 for 11% (*n* = 2), faster results with no wait time for an appointment with a specialist for 5% (*n* = 1), being able to get the treatment needed closer to home for 5% (*n* = 1), anonymity for 5% (*n* = 1), no long walk to the clinic for 5% (*n* = 1), and avoided long wait time once arriving at the appointment time for 5% (*n* = 1).

Among six patients who still required an in-person specialist visit after the eConsult, five of them were provided with useful information and next steps to manage their care while waiting to see the specialist (e.g. medication changes, tests needed, etc.).

About 66% of the patients (*n* = 19) stated that they would ask their PCP to use the eConsult service on their behalf in the future. However, just over half of patients (*n* = 16) (Table 2) agreed that the eConsult service was an acceptable alternative to face-to-face specialist consultations. Of those who believed eConsult service was not an acceptable alternative, three out of four believed a face-to-face meeting is better. One patient mentioned, “the specialist doesn’t see the patient face to face and might miss the diagnosis. Direct experience is better for a thorough diagnosis,“ as the reason of preferring a face-to-face meeting. No further information was provided by other patients regarding the reason they believe a face-to-face meeting is better.

**Fig. 2:**
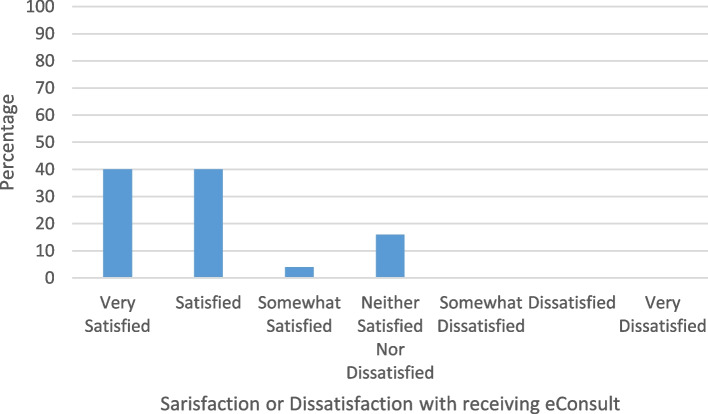
Satisfaction or dissatisfaction with the experience of receiving an electronic consultation in Manitoba

Overall, satisfaction or dissatisfaction with the experience of receiving an electronic consultation is shown in Fig. 2. About 80% of participants were satisfied or very satisfied with the experience of receiving an electronic consultation.

The last question was an open-ended question asking how this experience of receiving an electronic consultation could have been better for the patient. Overall, 24 patients answered the question. Table 3 shows all the answers.

**Table 3:** Patients’ answers (*n* = 24) to the question asking how the experience of receiving an electronic consultation could have been better for them

**Categories of patients’ answers**			
Positive comments	Request for improvement	Unique circumstances	Nothing to add
I like telephone call appointments. I was quickly booked for an in person as needed. Way better than taking the whole day off.	was not told in advance it was being done	I have a unique case, a joint condition called Ehlers Danlose Syndrome. It’s rarely researched, and as such, it’s common for doctors not to know what to do. Appointments regularly end with no new help or information. This is an issue with in-person doctor visits as well, and although I don’t know how to solve the issue, it remains to be a prevalent issue throughout my life.	It was great! No changes
It’s good	If someone had called me with my results. I had to call in to get my results		There’s nothing to complain about.
it was fine for me. but I do feel there is benefit with F2F. my case was a routine call.	Future ways of checking other body functions that could reduce the number of visitations in a clinic for non emergency situations		Na
I wish my doctor used eConsult previous to COVID	In person referral is better		Nothing
more convenient	I like face to face much better		All in all it works just fine for me the way it is
			Unsure
			None
			It couldn’t have been better
			I don’t think it could have been better
			Na
			Nothing to improve
			I don’t think it could have been better. I think it works in theory but in-person appointments can’t be replaced as sometimes there is a need for physical examination that can’t be done over a phone call.
			It was great

## Discussion

Our study demonstrated that the vast majority of patients were satisfied with the use of eConsult and think it as a useful and acceptable tool to access specialist advice. The vast majority responded they would ask their PCP to use the service again in the future. We also observed patients reporting from their perspective that saving money and time were perceived as major benefits of this type of asynchronous virtual care.

These results are consistent with other similar eConsult services in Canada [15]. A comparison between the results of the two provinces is illustrated in Additional file 4. One difference in our methodology was that in some of the identical questions, we allowed for respondents to choose, “unsure” which may have accounted for some of our slightly lower positive response rates.

A systematic review evaluating several international eConsult services demonstrated a satisfaction rate for receiving specialty advice in this manner ranges between 78 and 93% [12]. Our study found similarly high rates of satisfaction (80%) which indicates that patients perceive eConsult to be a useful tool for their care and this appears to be consistent across jurisdictions and health systems. With an aging and growing population in rural Manitoba [18] expansion of health services such as BASE eConsult may be key to improve access to care across the Province.

A related study conducted in Ontario by a similar service evaluated perspectives on wait times and using eConsultation as an alternative to a face-to-face visit. They found most patients thought wait times exceeded what they thought was ideal and 46% of patients thought that an eConsult would be an appropriate alternative to an in-person specialist visit [16]. In our study 55% of participants believed eConsult was an acceptable alternative to face-face visits with a specialist. It is uncertain if this percentage reflects a change in attitudes towards virtual care related to widespread adoption during related to the COVID-19 pandemic, natural change over time or differences in the sample population. In a multisite study in United States in 2016, 78.1% of the patient who received an eConsult as an alternative for face to face referral, chose the same service for the similar problem in the future. Patients with high PCP trust were more satisfied with the results of eConsult.

eConsult services are now common both in Canada and internationally [19]. In fact, in Canada eConsult is considered standard of care by the College of Family Physicians of Canada [20] and the Royal College of Physicians and Surgeons of Canada [21]. There is robust evidence that eConsult services are highly valued by PCPs [22], and this study adds to growing evidence that eConsult services are valued by patients. Currently efforts persist in several jurisdictions including Manitoba to equitably expand eConsult services so that all providers can access specialist advice this way and that patient care can be enhanced for those who need it the most.

In spite of the consistency with similar assessments in the literature, our study had several limitations. Firstly, our sample size is small and only conducted in a single jurisdiction, in an urban setting thus may not be generalizable to all settings and patient characteristics. It is possible that those who have to travel further and have more geographic barriers to care, might have felt more favorably to avoiding an in-person medical visit. Secondly, based on advice from our patient partners we included the option to answer “unsure” which did not allow for the identical comparison to similar surveys. For surveys conducted over the phone, it is possible the interviewer may have been able to explain some of the questions to avoid “unsure” answers but this would not have been available for those conducted electronically. Additionally, because the survey was conducted some time after the eConsult, it is possible there may be recall, perception or social desirability bias. We did not ask about several patient characteristics, nor why some questions may have been unanswered thus were we unable to assess the impact on our results.

The survey sample might be biased by self-selection since the patients themselves decided to answer the survey. Given the ethical considerations in conducting a study such as this, these are unavoidable.

## Conclusion

Overall, patients report favorable experiences and perceptions after having received specialty advice through eConsult and they believed it can be an effective and acceptable way to access care with many perceived benefits.

## Supplementary Information

**Additional file 1.** **Appendix A.** Patient Survey. 

**Additional file 2.** **Appendix B.** Clinic Participation Invite.

**Additional file 3: Appendix C.** Patient Invitation with Consent Disclosure Statement

**Additional file 4. **A comparison between participants’ perspective on eConsult services in Manitoba and Ontario. 

**Additional file 5. **Comparison of the results for those whose PCP asked them the questions that they wanted answered by specialist to those whose PCP did not offer that opportunity.

## Data Availability

The data that support the findings of this study are available on request from the corresponding author upon reasonable request. The data are not publicly available due to privacy or ethical restrictions. If there is any need, please contact the corresponding author at any time (corresponding author Alexander Singer*: Alexander.singer@umanitoba.ca).

## References

[1] How Canada Compares (2015). Results from the Commonwealth Fund 2014 International Health Policy Survey of older adults.

[2] Health Care in Canada (2012). 2012: A focus on wait times.

[3] Moir M, Barua B. Waiting your turn: wait times for health care in Canada, 2021 report. Fraser Institute. Cited October 19, 2022.

[4] Liddy C, Moroz I, Affleck E, Boulay E, Cook S, Crowe L (2020). How long are Canadians waiting to access specialty care? Retrospective study from a primary care perspective. Can Fam Physician.

[5] Waiting your turn: wait times for health care in Canada. Vancouver. 2021. Available from: https://www.fraserinstitute.org/categories/health-care-wait-times.

[6] World population review. 2022. Available from: https://worldpopulationreview.com/canadian-provinces/manitoba-population.

[7] Liddy C, Bello A, Cook J, Drimer N, Pilon MD, Farrell G (2019). Supporting the spread and scale-up of electronic consultation across Canada: cross-sectional analysis. BMJ Open.

[8] Keely E, Liddy C, Afkham A (2013). Utilization, benefits, and impact of an e-consultation service across diverse specialties and primary care providers. Telemed J E Health.

[9] Liddy C, Afkham A, Drosinis P, Joschko J, Keely E (2015). Impact of and satisfaction with a New eConsult Service: a mixed methods study of primary care providers. J Am Board Fam Med.

[10] Liddy C, Maranger J, Afkham A, Keely E (2013). Ten steps to establishing an e-consultation service to improve access to specialist care. Telemed J E Health.

[11] Liddy C, Drosinis P, Deri Armstrong C, McKellips F, Afkham A, Keely E (2016). What are the cost savings associated with providing access to specialist care through the Champlain BASE eConsult service? A costing evaluation. BMJ Open.

[12] Liddy C, Drosinis P, Keely E (2016). Electronic consultation systems: worldwide prevalence and their impact on patient care-a systematic review. Fam Pract.

[13] Ackerman SL, Dowdell K, Clebak KT, Quinn M, Shipman SA (2020). Patients assess an eConsult Model’s acceptability at 5 US Academic Medical Centers. Ann Fam Med.

[14] Gaye M, Mehrotra A, Byrnes-Enoch H, Chokshi D, Wallach A, Rodriguez L (2021). Association of eConsult implementation with Access to specialist care in a large Urban Safety-Net System. JAMA Health Forum.

[15] Joschko J, Liddy C, Moroz I, Reiche M, Crowe L, Afkham A (2018). Just a click away: exploring patients’ perspectives on receiving care through the Champlain BASETM eConsult service. Fam Pract.

[16] Keely E, Traczyk L, Liddy C (2015). Patients’ perspectives on wait Times and the Referral-Consultation process while attending a Tertiary Diabetes and Endocrinology Centre: is Econsultation an acceptable option?. Can J Diabetes.

[17] (CFHI) CFfHI (2017). Connected medicine workshop.

[18] Ashton B, LaBelle S, Mealy R, Wuttunee W (2022). State of Rural Canada.

[19] Liddy C, Joschko J, Guglani S, Afkham A, Keely E (2019). Improving equity of Access through Electronic Consultation: a case study of an eConsult Service. Front Public Health.

[20] (CFPC) TCoFPoC. Available from: https://www.cfpc.ca/en/policy-innovation/innovation-in-research-and-quality-improvement/practice-improvement-initiative.

[21] Canada RCoPaSo (2018). Statement of support for eConsult as standard of practice.

[22] Lee MS, Ray KN, Mehrotra A, Giboney P, Yee HF, Barnett ML (2018). Primary care practitioners’ perceptions of electronic Consult Systems: a qualitative analysis. JAMA Intern Med.

