# Supplementary material for: Evaluation of BASE eConsult Manitoba: patient perspectives on the use of electronic consultation to improve access to specialty advice in Manitoba
Source: BMC Health Serv Res. 2023 Feb 9;23:131. doi: 10.1186/s12913-022-08913-3 (PMC9909129; doi:10.1186/s12913-022-08913-3)
Supplement: Supplementary file 1 — Additional file 1. Appendix A. Patient Survey. [file 12913_2022_8913_MOESM1_ESM.docx]

**Evaluation of BASE eConsult MB: Patient Perspectives on the use of Electronic Consultation to Improve Access to Specialty Advice in Manitoba**

**Patient Survey**

**Clinic:**

**Patient Information:**

Age:

First 3 Digits of Postal Code:

**Patient Survey:**

1. Do you think that the eConsult service was useful in your situation?
2. Yes
3. No
4. Unsure
5. Do you think that the eConsult service is an acceptable way to access specialist advice?
6. Yes
7. No
8. Unsure
9. During the visit when your Primary Care Provider (Family Physician or Nurse Practitioner) sent the eConsult, were you expecting to be referred to a specialist for a face-to-face consultation?

a) Yes

b) No

1. Before the eConsult occurred, did your Primary Care Provider ask you what questions you wanted answered by the specialist?

a) Yes

b) No, (skip to question 5)

c) Unsure, (skip to question 5)

4a) If yes, were your questions answered fully by the eConsult?

- 1. Yes
  2. No
  3. I have not yet received my eConsult results

1. Do you think that the eConsult service is an acceptable **alternative** to face-to-face specialist consultations?
2. Yes
3. No , Please explain
4. Unsure
5. Would you ask your Primary Care Provider to use the eConsult service on your behalf in the future?

a) Yes

b) No

c) Unsure

1. Did you receive the results from your eConsult?

a) Yes

b) No

c) None of the above

7a) How long did you wait to learn the results of the eConsult?

- 1. I waited (in days)
  2. I did not learn results (skip to question 8)

7b) Was this wait time acceptable to you?

a) Yes

b) No, a wait time of would have been more acceptable

c) N/A

1. How was the eConsult advice provided to you?
2. New scheduled appointment
3. Existing scheduled appointment
4. Telephone call
5. Email or other electronic communication
6. No follow-up regarding eConsult
7. As a result of your Primary Care Provider’s use of the eConsult service, did you avoid an in-person specialist visit?
8. Yes, I avoided a visit to a specialist
9. No, the eConsult confirmed that I should see a specialist (skip to 10b)

9a) If yes, how did avoiding a specialist in-person visit impact you. Select all that apply:

- Avoided costs (travel, parking, child or eldercare)
- Avoided time off work
- Reduced my stress level or anxiety
- Other (please describe)

9b) If the eConsult confirmed the need for a specialist face-to face visit did the eConsult also provide useful information and next steps to manage your care while waiting to see the specialist? (e.g. medication changes, tests needed, etc.)?

1. Yes
2. No
3. Overall, how satisfied or dissatisfied were you with the experience of receiving an electronic consultation
4. Very dissatisfied
5. Dissatisfied
6. Neither satisfied or dissatisfied
7. Satisfied
8. Very satisfied

11) How could this experience have been better for you?

12) Are there any other comments you would like to make, about your experience with eConsult?
