# Supplementary material for: Evaluation of BASE eConsult Manitoba: patient perspectives on the use of electronic consultation to improve access to specialty advice in Manitoba
Source: BMC Health Serv Res. 2023 Feb 9;23:131. doi: 10.1186/s12913-022-08913-3 (PMC9909129; doi:10.1186/s12913-022-08913-3)
Supplement: Supplementary file 2 — Additional file 2. Appendix B. Clinic Participation Invite. [file 12913_2022_8913_MOESM2_ESM.docx]

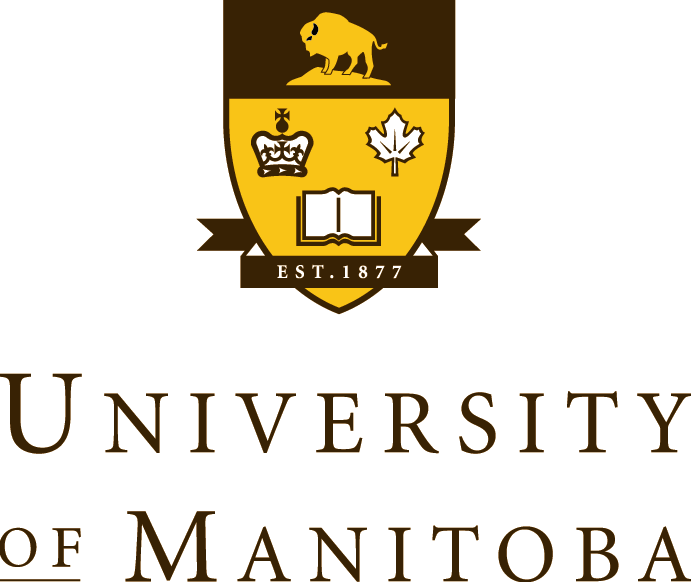


Faculty of Medicine


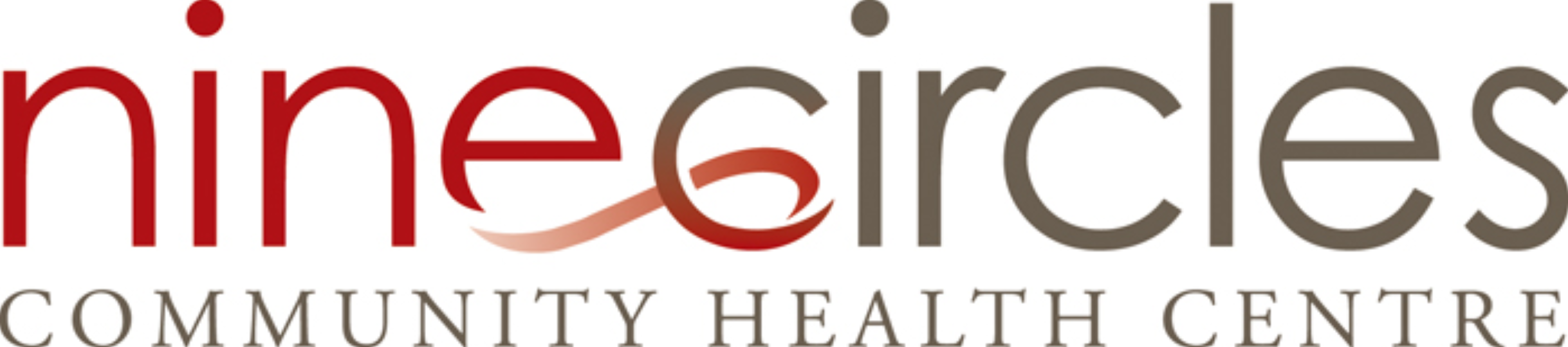

We would like to invite your clinic to participate in the study:

**Evaluation of BASE eConsult MB: Patient Perspectives on the use of Electronic Consultation to Improve Access to Specialty Advice in Manitoba**

This study’s objective is to gain understanding of patients’ experience with the use of electronic consultation to receive specialist advice on their care. A patient survey will be administered to patients who have received an eConsult in order to gain this understanding. You were specifically selected to participate because of your clinic’s participation in the BASE eConsult MB service and research study.

Our research team will support all participating clinics to identify eligible patient participants who have had an eConsult sent on their behalf. Our research team will then mail or email invitations to patients directly to ask them if they wish to participate in the patient survey online or over the phone.

I will attach a copy of the patient invitation and the survey for your review.

Should your clinic wish to participate, please contact the eConsult MB Project Manager, Kelly Brown who will confirm your interest and support your clinics participation.

Contact Information:

Kelly Brown: email: [mbeconsult@umanitoba.ca](mailto:mbeconsult@umanitoba.ca),Phone:

Best Regards,

| _________________________  Dr. Laurie Ireland  Lecturer, Family Medicine,  University of Manitoba  Medical Director,  Nine Circles Community  Health Centre  MB HIV Program,  Primary Care Lead | ________________________  Dr. Alexander Singer  Associate Professor,  Family Medicine,  University of Manitoba  Director,  Manitoba Primary Care  Research Network | _________________________  Dr. Luis Oppenheimer  Associte Professor,  Department of Surgery  University of Manitoba |
| --- | --- | --- |
