# Supplementary material for: Evaluation of BASE eConsult Manitoba: patient perspectives on the use of electronic consultation to improve access to specialty advice in Manitoba
Source: BMC Health Serv Res. 2023 Feb 9;23:131. doi: 10.1186/s12913-022-08913-3 (PMC9909129; doi:10.1186/s12913-022-08913-3)
Supplement: Supplementary file 3 — Additional file 3: Appendix C. Patient Invitation with Consent Disclosure Statement [file 12913_2022_8913_MOESM3_ESM.docx]

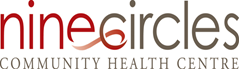


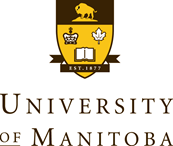
 Faculty of Medicine
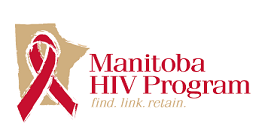


Dear XX Patient’s name,

You are being invited to participate in a University of Manitoba research study **“Evaluation of BASE eConsult MB: Patient Perspectives on the use of Electronic Consultation to Improve Access to Specialty Advice in Manitoba”.**

Your primary care provider has previously sent an electronic consultation or “eConsult” to a specialist to receive non-urgent advice on your health care treatment or management plan. The BASE eConsult research team would like to get your feedback on the use of eConsult in your health care and is asking you to consider completing a short 16 question survey.

The Study survey will take approximately 20 minutes to complete*.*

There is no risk to participation. Involvement in the study is voluntary and will not impact your care. There may be indirect benefits to you for participating in this study as the information may be used to inform future planning to support the ongoing availability and improvements to an electronic consultation service in Manitoba.

If you choose to participate, you will be offered a $10.00 Visa gift card

The survey information you provide will be kept confidential and does not include any personal health or identifiable information. If you would like to receive the gift card by mail, your contact information will be collected and remain separate from the survey results and be destroyed as soon as the card is mailed out. Participation is voluntary and you can choose to withdraw at any time.

If you have any questions about this research study, you may contact Dr. Laurie Ireland at lireland@ninecircles.ca or (204) 940-6000 or Dr. Alexander Singer at alexander.singer@umanitoba.ca or (204) 789-3314 or our research coordinator, Kelly Brown at (204) 940-6084.

If you choose to participate, the survey can be accessed in one of two ways:

1. By clicking the link and completing the survey online: [www.surveymonkeyXXXX.com](http://www.surveymonkeyXXXX.com)

OR,

1. You can call the research coordinator Kelly Brown and she will to ask you the survey questions over the phone. (204) 940 6084.

Thank you for your time,

Sincerely the BASE eConsult MB Research Team

Dr. Laurie Ireland MD CCFP,

Dr. Alexander Singer MB BAO BCh CCFP,

Dr. Luis Oppenheimer MD FRCPC
