# Supplementary material for: Evaluation of BASE eConsult Manitoba: patient perspectives on the use of electronic consultation to improve access to specialty advice in Manitoba
Source: BMC Health Serv Res. 2023 Feb 9;23:131. doi: 10.1186/s12913-022-08913-3 (PMC9909129; doi:10.1186/s12913-022-08913-3)
Supplement: Supplementary file 4 — Additional file 4. A comparison between participants’ perspective on eConsult services in Manitoba and Ontario. [file 12913_2022_8913_MOESM4_ESM.docx]

Additional file 4. A comparison between participants’ perspective on eConsult services in Manitoba and Ontario

|  | Manitoba | Ontario  (11) |
| --- | --- | --- |
| Characteristic | % | % |
| Do you think that the eConsult service was useful in your situation? ﻿  Yes  No  Unsure | 76  7  17 | 87  13  - |
| Do you think that the eConsult service is an acceptable way to access specialist advice?  Yes  No  Unsure | 83  3  14 | 100  -  - |
| Do you think that the eConsult service is an acceptable alternative to face-to-face specialist consultations?  Yes  No  Unsure | 55  14  28 | 70  3  27 |
| Would you ask your Primary Care Provider to use the eConsult service on your behalf in the future?  Yes  No  Unsure | 66  7  17 | 83  3  13 |
