# Supplementary material for: Evaluation of BASE eConsult Manitoba: patient perspectives on the use of electronic consultation to improve access to specialty advice in Manitoba
Source: BMC Health Serv Res. 2023 Feb 9;23:131. doi: 10.1186/s12913-022-08913-3 (PMC9909129; doi:10.1186/s12913-022-08913-3)
Supplement: Supplementary file 5 — Additional file 5. Comparison of the results for those whose PCP asked them the questions that they wanted answered by specialist to those whose PCP did not offer that opportunity. [file 12913_2022_8913_MOESM5_ESM.docx]

Additional file 5. Comparison of the results for those whose PCP asked them the questions that they wanted answered by specialist to those whose PCP did not offer that opportunity
